# Effectiveness of TachoSil as Sealant in Lymphatic Leakage of Breast Cancer With Axillary Dissection

**DOI:** 10.1155/ijbc/3765406

**Published:** 2024-12-31

**Authors:** ArshadUllah Khan, Loai Albinsaad, Mohammed Alessa, Alghaydaa Fouad Aldoughan, Ammar Jaafar Alsalem, Noof Khalid Almukhaimar, Abdulrahman Ahmed Alghamdi, Watan Abdulla Alsahlawi, Batool Abdullah Alahmary

**Affiliations:** ^1^Oncology and Breast Oncoplastic Surgery, AlAhsa Hospital, Al-Ahsa City, Eastern Province, Saudi Arabia; ^2^Department of Surgery, College of Medicine, King Faisal University, Al-Ahsa City, Eastern Province, Saudi Arabia; ^3^College of Medicine, King Faisal University, Al-Ahsa City, Eastern Province, Saudi Arabia

**Keywords:** axillary dissection, breast cancer, fibrin sealant, hemostatic agent, lymphocele

## Abstract

**Objectives:** This study is aimed at evaluating the effectiveness of TachoSil in controlling lymphatic leakage in breast cancer patients undergoing axillary dissection. By examining its ability to reduce postsurgical lymphatic drainage, the study will assess its impact on complications like seroma formation, recovery time, and overall patient outcomes, including quality of life and reduced healthcare costs.

**Methods:** Breast cancer patients treated in the Department of Surgical Oncology at King Abdulaziz Medical City were enrolled to receive either TachoSil or undergo drain placement after axillary dissection. Repeated measures multivariate analysis of variance (MANOVA) was used to observe the difference in lymphatic drainage volume over time considering other covariates, such as age, sex, family history, neoadjuvant chemotherapy (NAC), and stage.

**Results:** The TachoSil group showed significantly lower lymphatic drainage volumes at 24 h (106.5 ± 11.3) than the control group (141.7 ± 13.0) (*p* < 0.001). There were no significant differences in lymphatic drainage volume at 3 days (*p* = 0.176) and 7 days (*p* = 0.091). However, at 10 days, the TachoSil group exhibited significantly lower lymphatic drainage volume (19.9 ± 6.1) than the control group (44.5 ± 9.2) (*p* < 0.001). Repeated measures MANOVA showed a statistically significant difference in lymphatic drainage over time, with a moderate effect (*p* < 0.001).

**Conclusion:** The findings suggest that TachoSil sealant effectively reduces early postoperative lymphatic drainage volume and maintains lower drainage rates up to 10 days following axillary dissection in breast cancer patients. The use of TachoSil sealant may have potential benefits in reducing the incidence of complications associated with lymphatic drainage and improving patient outcomes.

## 1. Introduction

Breast cancer (BC) is one of the most prevalent cancers in women [1]. In 2020, it was linked to 15.5% of cancer deaths worldwide, 24.5% of all cancer diagnoses among women, and an approximately 21.8% prevalence in Saudi women [2, 3].

Previous studies have indicated that, in many cases, axillary dissection is an essential part of the management of BC [1]. Following axillary dissection, postoperative lymphatic leakage as a lymphocele is possible, which is usually managed by drainage [4]. This can cause pain, infection, and necrosis and can increase the number of hospitalization days [5]. Another postoperative morbidity that has been reported in 15%–85% of cases is seroma. Seroma development is influenced by a variety of variables, such as the size of the lymphatic interruption following surgery, a history of prior biopsies, the size of the breast and the residual cavity, the surgical approach used (mastectomy or lumpectomy), the use of electrocautery with a diathermy device, the interval of suction drainage, and cicatrization (the process of scar tissue formation during wound healing) [1]. Seroma is one of the most common complications that may cause the surgical wound to dehisce and the skin flaps to necrotize, which would increase the risk of infection and the need for a second procedure. It may also hinder the recovery of upper limb movement and postpone the initiation of any adjuvant therapies [6]. The pathophysiology for seroma formation seems to be multifactorial along with surgery itself [7]. Many methods for seroma prevention have been reported, but unfortunately, no single method is reliably effective [8]. For example, the implementation of continuous suction drainage [9], the topical application of tetracyclines [10], external compression bandages [11], and the use of ultrasound-assisted dissectors [12] are noteworthy. Furthermore, fibrin sealant has shown promising results due to its adhesive and hemostatic qualities, as well as its potential to stimulate tissue regeneration [13]. Closure of the dead space (quilting), however, was consistently found to be significantly effective [14]. The incidence of clinically significant seroma and the quantity of aspirations are both markedly reduced by quilting. There has been a noticeable drop in surgical site infection, which has more than halved the requirement for antibiotic therapy; one of the useful techniques for preventing seroma is quilting [14].

TachoSil is a collagen sealant patch coated with fibrinogen and thrombin. Applying it to the target area triggers the conversion of fibrinogen into fibrin, which is the last phase of the coagulation process. Thus, TachoSil is considered an alternative to the standard techniques used to control bleeding and leakage [15]. According to previous reports, it can be used for hemostasis during surgery [16]. For example, in some studies, TachoSil was used to control bleeding during kidney tumor resection [17, 18]. In addition, experiments conducted to evaluate the effectiveness of TachoSil in minimizing leakage from colorectal anastomoses in animals showed a reduction in leakage [19].

Another study investigated the use of TachoSil in cases of gynecologic malignancy following inguinofemoral lymphadenectomy to reduce the incidence of postoperative lymphatic leakage and the need for drainage [20]. TachoSil has also been shown to be useful in lowering the rate of lymphoceles following pelvic lymphadenectomy [21]. A study conducted in Saudi Arabia found that the application of fibrin sealant following inguinal lymphadenectomy resulted in a reduction in the formation of seroma, the amount and duration of drainage, as well as the length of hospital stay [16].

In a study conducted in Spain, the use of TachoSil after axillary dissection was effective in preventing symptomatic lymphocele, reducing drainage volume and duration, and reducing the length of hospital stay [5]. The use of TachoSil for preventing seroma and reducing leakage after axillary dissection has also been shown to be successful [22]. However, a trial conducted in 2018 showed that TachoSil was not effective in reducing the volume of drainage, and its use was accompanied by an increase in the duration of hospital stays [23].

Chang et al. [24] found that, regardless of the number of lymph nodes removed, the use of fibrin sealants was effective in patients undergoing axillary dissection. Although fibrin sealants did not prevent the incidence of postoperative lymphatic leakage after axillary dissection, they reduced the duration and volume of drainage [24, 25] and the duration of hospitalization [25].

Even though previous studies have suggested that fibrin sealants may decrease the chance of seroma, few trials have been conducted; therefore, the findings are still preliminary [24]. However, the use of cyanoacrylate glue in conjunction with a closed suction axillary drain appears to be linked to a decrease in the number of days that the axillary drain remains in place as well as a decrease in postoperative infections [6]. The aim of this study was to evaluate the use of TachoSil as a sealant for lymphatic leakage in BC patients with axillary dissection.

## 2. Methodology

### 2.1. Sample Size

A total of 66 BC patients undergoing axillary dissection were enrolled in this parallel-group study to evaluate the efficacy of TachoSil in minimizing lymphatic drainage compared to standard drain placement. The study was conducted with an allocation ratio of 1:1, meaning that patients were randomly assigned to one of two groups: one group received TachoSil, while the other group underwent traditional drain placement without TachoSil. Patients were recruited from the Department of Surgical Oncology at King Abdulaziz Medical City.

#### 2.1.1. Inclusion and Exclusion Criteria

All BC patients requiring axillary dissection, regardless of whether they underwent conservative breast surgery or modified radical mastectomy, were eligible to participate. Axillary dissection is a surgical procedure where lymph nodes are removed from the underarm to assess the spread of cancer. However, specific exclusion criteria were applied to ensure a uniform patient population.

### 2.2. Exclusion Criteria

Patients with recurrent BC were excluded to prevent any confounding factors related to prior treatments or surgeries. Patients with ductal carcinoma in situ (DCIS), a noninvasive form of BC, were also excluded as these cases often do not require extensive axillary dissection. BCs that did not necessitate axillary clearance were not included in the study, as the primary focus was on those who required lymph node removal.

### 2.3. Study Design and Procedure

Patients were divided into two groups of 33.

Group 1 (TachoSil group): In this group, 33 patients received TachoSil, a hemostatic agent used as a sealant following axillary dissection. The purpose of TachoSil is to reduce lymphatic leakage by forming a barrier at the surgical site, promoting faster sealing of lymphatic vessels.

Group 2 (control group—drain placement only): In the second group, 33 patients underwent the standard treatment protocol of drain placement alone, without the use of TachoSil. Drains are typically inserted to manage lymphatic discharge and prevent fluid accumulation (seroma).

### 2.4. Data Collection and Measurements

To assess the effectiveness of TachoSil in reducing postoperative lymphatic leakage, the volume of fluid discharge was measured at multiple time points postsurgery. Drain output was recorded at 24 h, 3, 7, and 10 days following the procedure in both groups. These drain readings were used to track the amount of lymphatic fluid being discharged, which is a key indicator of how well the surgical site is healing and the effectiveness of the intervention in reducing leakage.

The study followed a strict protocol, with no changes made to the eligibility criteria after the trial commenced. This ensured consistency in patient selection and data collection throughout the study.

The comparison between the two groups is aimed at determining whether TachoSil significantly reduced the volume of lymphatic discharge, potentially leading to shorter hospital stays, reduced risk of complications like seroma, and faster recovery for patients. The results from the drain readings would provide critical data on the effectiveness of TachoSil as a sealant in postoperative care for BC patients undergoing axillary dissection.

### 2.5. TachoSil

After axillary dissection up to Level 2, we placed two patches of the TachoSil after activating with normal saline over the axilla. The yellow part of the TachoSil paste over the axilla where sealing effect is required. After placing the TachoSil, we placed a drain to observe output and closed the wound in layers.

### 2.6. Postoperative Care

Postoperatively, we observed the drain out in 24 h, 3, 7, and 10 days, postoperatively.

When a drain output of less than 30 mL was recorded, then we removed the drain. We advised adjustable compression wrap in selected patients because we want to observe the effect of TachoSil on lymphatic leakage.

No changes in eligibility criteria after trial commencement.

### 2.7. Statistical Analysis

Taking the confidence interval of 95% and the power of the study of 80%, the mean and standard deviation of total volume drained as 326.69 mL ± 167.5 in the TachoSil group and as 473.89 mL ± 230 in the control group, we get the sample size of 30 in each group. Also, for the total volume drained each day from 1 to 5, the mean and standard deviation were used from the same parent study [5], and the highest sample size was calculated from Day 1, that is, 103.33 mL ± 67.5 and 129.24 mL ± 77.5; sample size obtained was 124. Therefore, we will use the highest sample size, that is, 124 in each group for recruiting our study participants. The data were collected, reviewed, and then analyzed using the Statistical Package for the Social Sciences (SPSS), Release 26 (SPSS Inc., Chicago, IL). All analyses were performed with two-sided tests; *p* < 0.05 was considered significant. The mean was calculated for demographic data such as gender distribution and age, as well as in the drain readings after 24 h, 3, 7, and 10 days. Repeated measures MANOVA was used to observe the difference in lymphatic drainage over time considering other covariates, such as sex and age. The random allocation sequence was generated by lottery method by computer software using principal investigation.

## 3. Results

The current study included 66 BC patients who underwent axillary dissection. The periods of recruitment and follow-up were between June 2015 and June 2017. The trial was completed in the hospital, and the primary investigator left the job so data analysis was stopped. TachoSil is commonly used in haemostasis so it has no harmful effects on the human body.

The lymphatic drainage was measured after axillary dissection in two groups, where 33 patients were given TachoSil as a sealant and another 33 patients were not given TachoSil (control group). The mean age of the patients was 53.4 ± 10.8 years, which did not show any statistically significant differences between the two groups (*p* = 0.516). The age distribution showed that 28 (42.4%) were between 51 and 60 years, which also showed statistically significant differences between the two groups (*p* = 0.775). Approximately 64 (97.0%) were female patients, and there were no significant differences between the two groups (*p* = 0.151). A family history of BC was reported in 57 (86.4%) patients, and neoadjuvant chemotherapy (NAC) was equally distributed between the two groups. Only Stage 2 (56.1%) and Stage 3 (43.9%) BCs were found in our sample, which did not show any statistically significant difference between the two groups (Table 1).

We observed the drain out in 24 h, 3, 7, and 10 days, postoperatively.

**Figure 1:**
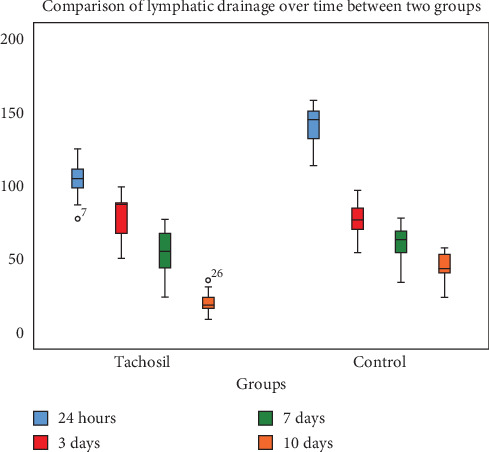
Comparison of lymphatic drainage overtime between two groups.

When the drain output recorded less than 30 mL, we remove the drain. We advised adjustable compression wrap in selected patients because we want to observe the effect of TachoSil on lymphatic leakage. The lymphatic drainage after 24 h was found to be significantly lower in the TachoSil group (106.5 ± 11.3) than in the control group (141.7 ± 13.0) (*p* < 0.001). There were no statistically significant differences observed in lymphatic drainage between the two groups after 3 days (*p* = 0.176) and 7 days (*p* = 0.091). However, a statistically significant difference was observed in lymphatic drainage after drainage after 10 days, where the TachoSil group showed lower values (19.9 ± 6.1) than the control groups (44.5 ± 9.2) (*p* < 0.001) (Table 2, Figure 1).

Repeated measures MANOVA was performed to determine the differences in lymphatic drainage over time while considering other covariates (independent variables) (Table 3). A statistically significant effect was found in lymphatic drainage over time with a moderate effect (*F*(1.58) = 59.24, *p* < 0.001, *η*2 = 0.50).

## 4. Discussion

In recent decades, the surgical approach to BC has shifted from more extensive techniques, such as radical mastectomy, to less invasive options like breast-conserving surgery. Similarly, the management of the axilla has significantly transformed, moving from routine axillary dissection to sentinel lymph node biopsy (SLNB) [26]. Axillary lymph node dissection has traditionally been the standard treatment for patients with a positive sentinel lymph node. Studies have indicated that a common complication associated with these treatments is the development of seromas. Prolonged axillary lymphatic drainage following axillary dissection for BC is not a life-threatening complication. However, it is still the leading cause of extended hospital stays and higher medical expenses. In women with BCs, excessive lymphatic drainage is a problem that can delay the start of adjuvant treatment and often necessitates additional procedures. There is not yet a standardized protocol for preventing lymphatic drainage complications following pelvic and/or para-aortic lymphadenectomy, despite the widespread use of techniques such as peritoneal repair, lymphangiography, and fibrin sealants. Thus, the current study evaluated the effectiveness of TachoSil in minimizing lymphatic drainage after axillary dissection. The TachoSil is specially designed for sealing bleeding and lymphatics and contains active components such as fibrinogen and thrombin, which are present in the yellow side of the patch. The yellow part is an active part, and TachoSil should be pasted from the yellow side on the raw area. When the active side comes into contact with fluids (such as blood, lymph, or saline solution), the fibrinogen and the thrombin are activated and form a fibrin network, and it causes a sealing effect over the bleeding area and lymphatic leakage. The findings of this study showed that lymphatic drainage was significantly less in patients who were given TachoSil as a sealant compared to the control group. TachoSil promotes more stable fibrinogen clot formation in the last phase of coagulation [27]. TachoSil not only prevents further bleeding but also forms an adhesive barrier. Because of its hemostatic and adhesive characteristics, TachoSil may facilitate less traumatic closure of injured lymphatic vessels, hence decreasing lymphatic drainage [28]. In contrast to liver, lung, and vascular surgeries, where there are investigations emphasizing its efficacy as a hemostatic product favoring the sealing of the intervened regions, there are only a few international studies assessing the use of TachoSil as a sealant in BC patients after axillary dissection, with very different and disparate results [29].

Research conducted on animals has shown that fibrin gel can be useful in lymphadenectomies after mastectomy [30]. Clinical studies on humans have shown contradictory findings, with some showing the products as helpful while others showing no impact. The current study findings showed that TachoSil was beneficial in lowering the total volume of lymphatic drainage following axillary dissection, regardless of BC stage or surgical type. Although the use of fibrin-made sealant was shown to inhibit lymphatic outflow in rats according to Lindsey et al. [31], clinical studies have shown mixed outcomes. Five studies [32–36] have examined how fibrin sealant affects drain times and total drain output. Three of the investigations found that fibrin glue decreased both the rate and duration of lymphatic outflow in the axilla [32–34], whereas the other two found no impact [35, 36]. Furthermore, a meta-analysis of multiple trials concluded that while the data seemed to show lower total drain output and a shorter drainage time in subjects receiving fibrin sealant, those findings were not statistically significant [37]. Because of the effect of fluid from the mastectomy site or the heterogeneity of surgery, it is difficult to adequately analyze axillary lymphatic drainage in those studies that involved virtually only mastectomy patients or varied instances seldom involving lumpectomy. Fibrin sealants have been shown to shorten the hospital stays of women after BC surgery and decrease the amount of serum secreted from the axilla [38, 39]. Fibrin sealants have been shown in several prior studies to minimize lymphatic fluid drainage volume and shorten hospital stays following BC surgery, including axillary lymph node dissection [5, 38]. Felsingerová, Minář, and Weinberger [40] found that pelvic lymph node dissection as part of a staging procedure would result in a longer hospital stay and increased patient pain due to the high volume of lymphatic flow. However, we did not account for hospital stay duration in our analysis because patients in our country are typically only released once pathology is verified. With most costs covered by health insurance, most BC patients in Saudi Arabia are taken care of in hospitals until their wounds have completely healed and they receive their first round of chemotherapy. There is a more significant association between the date of pathologic confirmation or the day of complete suture removal and the beginning of chemotherapy and the length of hospital stay [41].

Many researchers have reported the efficacy of TachoSil in preventing lymphocele development following laparoscopic staging surgery with pelvic lymph node dissection [21], although there have also been trials with poor outcomes. TachoSil was shown to be ineffective by Achouri et al. [42] in preventing lymphocele development during lymphadenectomy for pelvic gynecologic cancer. The most common cause of lymphocele is trauma to the lymphatic system. When comparing lymphocele rates, it is important to account for a variety of factors, such as the lymphocele detection technique, the follow-up period, the energy source used, the surgical technique, the patient's body mass index, and the number of lymph nodes removed [43]. The aforementioned variables and their associations with lymphocele occurrences have been the subject of several reports in the published literature [42, 43]. Köhler et al. [44] found that tattoos discolored lymph nodes in 33% of patients, and these patients subsequently had infected lymphoceles on the affected side. In our study, we did not find any significant influence of age, sex, family history of BC, or NAC on lymphatic drainage after BC surgery.

There are some limitations in our study. First, due to the limited size of the sample, we were unable to make any conclusions on the efficacy of fibrin sealants, although the lymphatic drainage seemed to be less. A larger sample size would be ideal, in our opinion, for verifying our findings. Second, the two sets of data were not directly comparable since they were collected at separate times. For this reason, it is clinically more necessary to assess TachoSil's effect on the frequency of symptomatic lymphoceles, following axillary lymphadenectomy. As a result, we propose to conduct multicenter randomized clinical research to evaluate the efficacy of a collagen-fibrin patch in the prevention of symptomatic lymphoceles in women who will undergo axillary dissection to undergo mastectomy. Despite these caveats, the results of our study suggest that TachoSil may be useful for minimizing lymphatic drainage in BC patients following axillary lymphadenectomy.

## 5. Conclusion

The study shows that TachoSil is an effective solution for minimizing lymphatic drainage in BC patients after axillary lymphadenectomy. The excessive drainage resulting from axillary dissection is a major concern, as it leads to increased healthcare costs and longer hospital stays. By minimizing lymphatic discharge, patients can experience physical and emotional relief, leading to an improvement in their quality of life. Therefore, TachoSil can be confidently recommended as a sealant to minimize lymphatic drainage and reduce hospital stays for at-risk patients.

## Figures and Tables

**Table 1:** Baseline characteristics of the patients based on two groups.

			**Groups**		**Total**	**p** ** value**
			**TachoSil**	**Control**		
	**Mean (SD)**		**54.3 (12.2)**	**52.5 (9.5)**	**53.4 (10.8)**	**0.516**
Age	≤ 40 years	*N*	4	4	8	0.775
		%	50.0%	50.0%	12.1%	
	41–50 years	*N*	9	7	16	
		%	56.30%	43.80%	24.2%	
	51–60 years	*N*	12	16	28	
		%	42.90%	57.10%	42.4%	
	≥ 61 years	*N*	8	6	14	
		%	57.10%	42.90%	21.2%	

Gender	Female	*N*	33	31	64	0.151
		%	51.60%	48.40%	97.0%	
	Male	*N*	0	2	2	
		%	0.0%	100.0%	3.0%	

Family history	No	*N*	33	24	57	0.001
		%	57.90%	42.10%	86.4%	
	Yes	*N*	0	9	9	
		%	0.0%	100.0%	13.6%	

NAC	No	*N*	17	17	34	1.000
		%	50.0%	50.0%	51.5%	
	Yes	*N*	16	16	32	
		%	50.0%	50.0%	48.5%	

Stage of breast cancer	Stage 2	*N*	19	18	37	0.804
		%	51.40%	48.60%	56.1%	
	Stage 3	*N*	14	15	29	
		%	48.30%	51.70%	43.9%	

Abbreviations: NAC, neoadjuvant chemotherapy; SD, standard deviation.

**Table 2:** Comparison of lymphatic drainage overtime between two groups.

	**Group**	**N**	**Mean**	**Standard deviation**	**p** ** value**
24 h	TachoSil	33	106.5	11.3	< 0.001
	Control	33	141.7	13.0	
3 days	TachoSil	33	81.1	13.9	0.176
	Control	33	77.1	9.5	
7 days	TachoSil	33	54.8	15.1	0.091
	Control	33	60.6	12.2	
10 days	TachoSil	33	19.9	6.1	< 0.001
	Control	33	44.5	9.2	

**Table 3:** Repeated measures MANOVA model.

**Source**	**Type III sum of squares**	**df**	**Mean square**	**F**	**p** ** value**	**Partial eta squared**
Intercept	9943.188	1	9943.188	42.74	< 0.001	0.424
Gender	1.441	1	1.441	0.006	0.938	0.000
Age	275.439	1	275.439	1.184	0.281	0.020
Diagnosis	285.881	1	285.881	1.229	0.272	0.021
Family history	121.946	1	121.946	0.524	0.472	0.009
Stage	212.57	1	212.57	0.914	0.343	0.016
NAC	236.399	1	236.399	1.016	0.318	0.017
Group	13,781.838	1	13,781.838	59.24	< 0.001	0.505
Error	13,493.375	58	232.644			

Abbreviations: df, degrees of freedom; NAC, neoadjuvant chemotherapy.

## Data Availability

The data that support the findings of this study are available upon request, but access is subject to certain restrictions. Due to privacy and ethical concerns, access to the data can only be granted to qualified researchers who meet specific criteria for data access. Researchers interested in accessing the data may contact Dr. Loai Saleh Albinsaad at Dr.loaisaleh@gmail.com to inquire about the procedures for data access, including the criteria and requirements for eligibility. Please note that access to the data may require the completion of a data access agreement and approval from the relevant ethics committee or institutional review board.
